# Acquired uterine arteriovenous malformation: efficacy of the use of absorbable haemostatic gelatin in uterine artery embolisation

**DOI:** 10.1007/s10140-025-02386-7

**Published:** 2025-09-10

**Authors:** Thay Hui Tan, Kenneth K. Lau

**Affiliations:** 1 Monash Imaging, Monash Health, VIC Clayton, Australia; 2 School of Clinical Sciences, Faculty of Medicine, Nursing and Health Sciences, Monash University, VIC Clayton, Australia; 3 Sir Peter MacCallum department of Oncology, University of Melbourne, VIC Parkville, Australia

**Keywords:** Uterine arteriovenous malformation, Uterine artery embolisation

## Abstract

**Purpose:**

To evaluate the efficacy and complications of absorbable haemostatic gelatin uterine artery embolisation for symptomatic acquired uterine arterio-venous malformation (UAVM).

**Methods:**

All the adult female patients who had acute urogenital bleeding due to UAVM confirmed on ultrasound and received uterine artery embolisation (UAE) for UAVM in a tertiary institution between January 2000 and October 2024 were included. Patients who had UAE for other causes were excluded. Causes of UAVM, embolic agents used, procedural success, recurrent genital bleeding, and complications like pulmonary embolism and groin bleeding were recorded.

**Results:**

Seven female patients (mean age: 34 years) with 8 UAE procedures were included, with three postpartum and four after miscarriages. The mean length of follow-up after UAE was 50 months. Absorbable gelatin was used in six patients, and polyvinyl alcohol (PVA) particles were used in one patient. 5/6 patients (83.3%) had successful UAE with absorbable haemostatic gelatin. There were no procedure-related complications, including pulmonary embolism and uterine infarcts. Only one patient required a repeated UAE 33 days later for recurrent vaginal bleeding, which required sodium tetradecyl sulphate injection and microcoils during embolisation.

**Conclusion:**

Acquired UAVM is very rare but life-threatening. Absorbable haemostatic gelatin, a temporary embolic agent, appears safe and effective in treating UAVM with uterine preservation. It eliminates the potential risk of uterine infarction that might occur with permanent embolic agents.

**Graphical Abstract:**

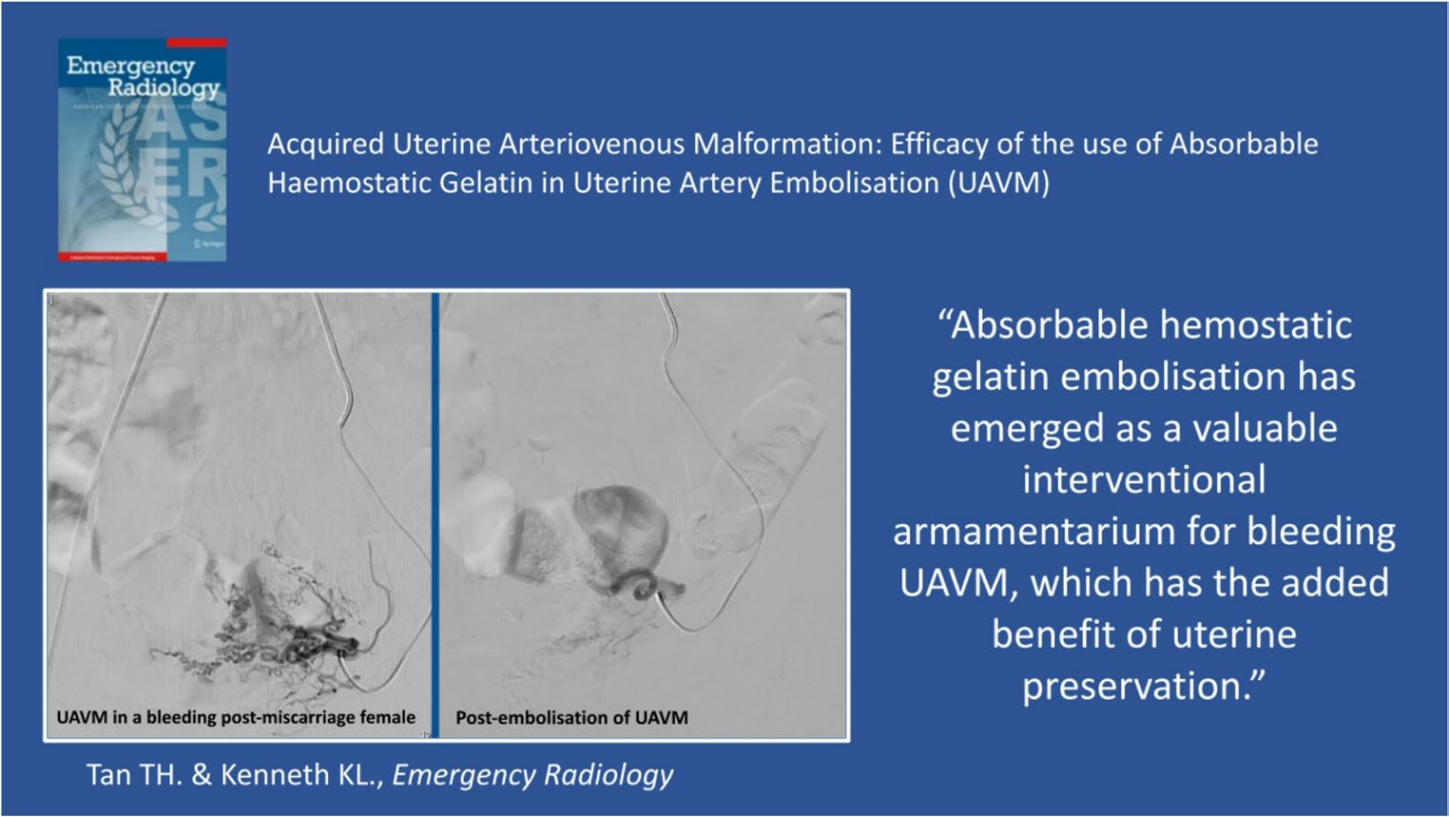

## Introduction

Acquired uterine arteriovenous malformation (UAVM) is very rare but life-threatening that occurs after delivery, miscarriage, or instrumentation such as dilation and curettage (D&C) in reproductive female patients. The incidence of acquired uterine AVM is 1–2% of all genital and intraperitoneal bleeding incidents [1]. Acquired UAVM is characterised by a fistula between uterine artery branches and myometrial venous plexus, leading to abnormal blood flow and, in many cases, associated with severe haemorrhage [2].

Transvaginal doppler ultrasound is the initial diagnostic tool for UAVM due to its ability to identify abnormal vascular patterns and high-velocity turbulent blood flow [3]. Historically, hysterectomy was considered the definitive treatment option for symptomatic uterine AVMs. However, this surgical approach carries drawbacks such as irreversible loss of fertility, increased recovery time, and postoperative complications [4]. Uterine artery embolisation (UAE) has now emerged as a treatment option for UAVM. It is the recommended first-line treatment given its being relatively minimally invasive and the ability for the patient to retain fertility in women of childbearing age [5].

Various embolic agents have been used successfully, including temporary absorbable haemostatic gelatin sponge (Gelfoam) and other permanent embolic agents such as Polyvinyl alcohol (PVA) particles, sodium tetradecyl sulphate, and mechanical coils [6]. The use of permanent embolic agents poses the risk of ischaemic complications as it completely blocks the blood flow for an extended period. Conversely, absorbable haemostatic gelatin is a temporary hygroscopic agent, allowing the concentration of platelets and clotting factors and providing additional mechanical haemostatic action through compression [7]. The theoretical advantage is that it naturally disintegrates or absorbs within 4 to 6 weeks, restoring normal blood circulation to the tissue after achieving the initial haemostatic desired effect [8]. The aim of this retrospective case series was to evaluate the efficacy and complications of absorbable haemostatic gelatin UAE for symptomatic acquired uterine AVM.

## Methods

All adult female patients from the Accident and Emergency department (A&E) who had acute urogenital bleeding due to UAVM confirmed on ultrasound and received UAE for UAVM in a tertiary institution between January 2000 and October 2024, identified on the Picture Archiving and Communication System (PACS), were included. Patients who had UAE for other causes were excluded. The patients’ ages, causes of UAVM, details of the UAE procedures, including attending interventional radiologists, types of embolic agents, and any immediate complications were noted. Post-procedural clinical history and follow-up for procedural success, recurrent genital bleeding, and complications were also recorded.

### Procedure

The procedures were performed in the angiography suite. A 5-Fr sheath was introduced into the right common femoral artery of the patient. The internal iliac arteries were initially selected with a Cobra catheter and glide wire. The uterine branches of bilateral internal iliac arteries were then supra-selected with a microcatheter, followed by the selective uterine angiogram to identify the bleeding uterine AVM. The AVM was then embolised with absorbable haemostatic gelatin slurry or other embolisation materials such as PVA particles or coils. Absorbable haemostatic gelatin was prepared by cutting the gelatin sheet into cube-shaped pieces and placing them in a 10 mL syringe. This was subsequently mixed with 5 mL of saline and 5 mL of contrast in another 10 mL syringe through a 3-way stopcock. The mixture was agitated between the two syringes until a desirable slurry consistency was achieved. Post-embolisation angiography was undertaken to demonstrate a complete occlusion of the abnormal AVM. The femoral artery vascular access was closed with a plug-based closure device or manual compression.

## Results

A total of seven A&E female patients (mean age: 34 years; age range: 27 to 43 years) were included in our study. All these patients presented to the Emergency Department with recurrent or ongoing vaginal bleeding, which was persistent despite conservative management. The diagnosis of UAVM was confirmed on the uterine Doppler ultrasound. The underlying cause of their UAVM was identified as postpartum-related in 2 patients and post-miscarriage in 5 patients. Among the post-miscarriage group, 4 patients had undergone unsuccessful D&C procedures that failed to stop the bleeding before undergoing UAE. In the postpartum group, 1 patient had a failed D&C preceding UAE. There was no exclusion, as no A&E patients had UAE for other bleeding causes.

**Fig. 1:**
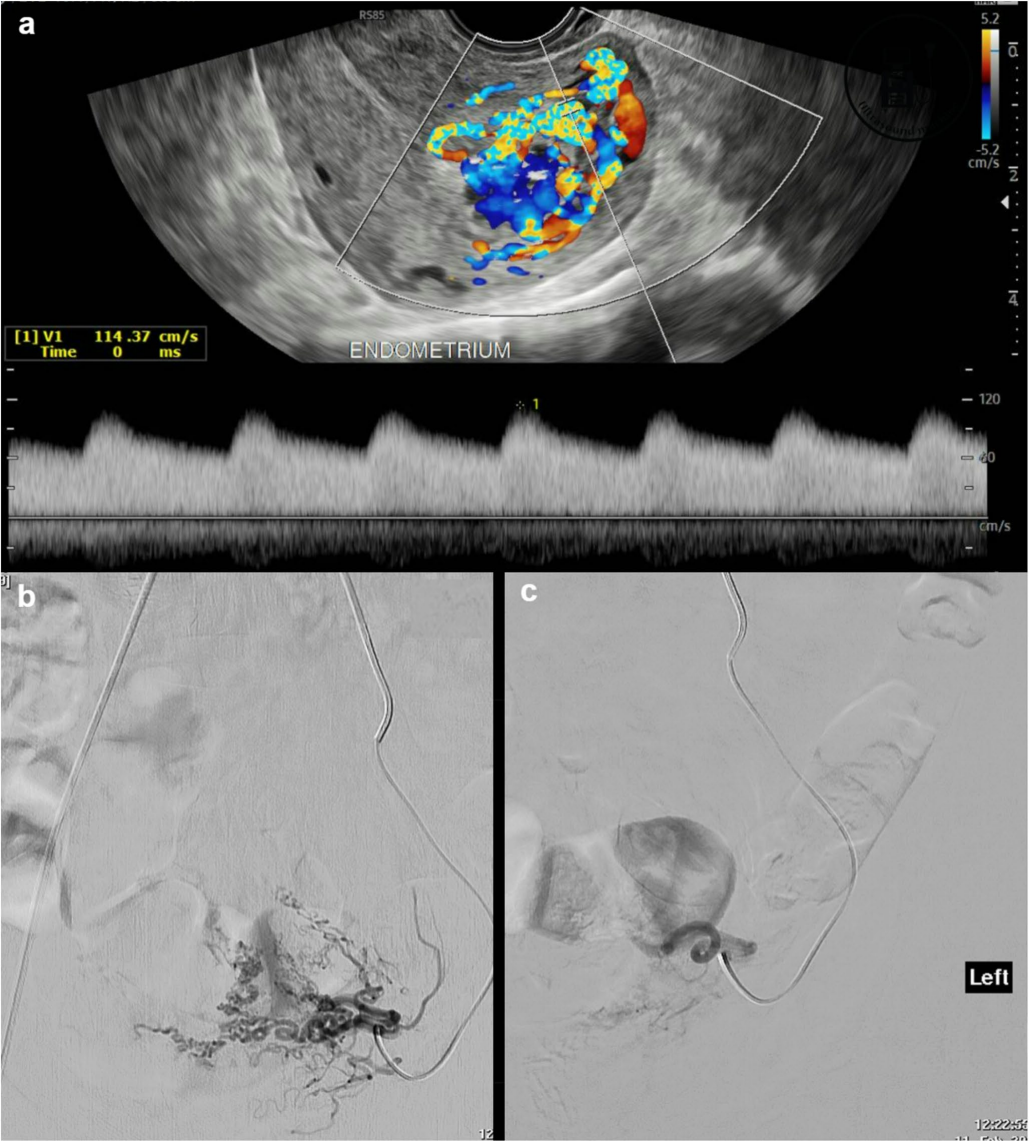
A 37-year-old female presented with vaginal bleeding after miscarriage. Pelvic ultrasound (**a**) confirmed the UAVM. Selective left uterine artery angiogram with a micro-catheter confirmed a cluster of tortuous and hypertrophied abnormal vessels (**b**), confirming the ultrasound finding. Successful UAVM embolisation with absorbable haemostatic gelatin was achieved (**c**)

**Fig. 2:**
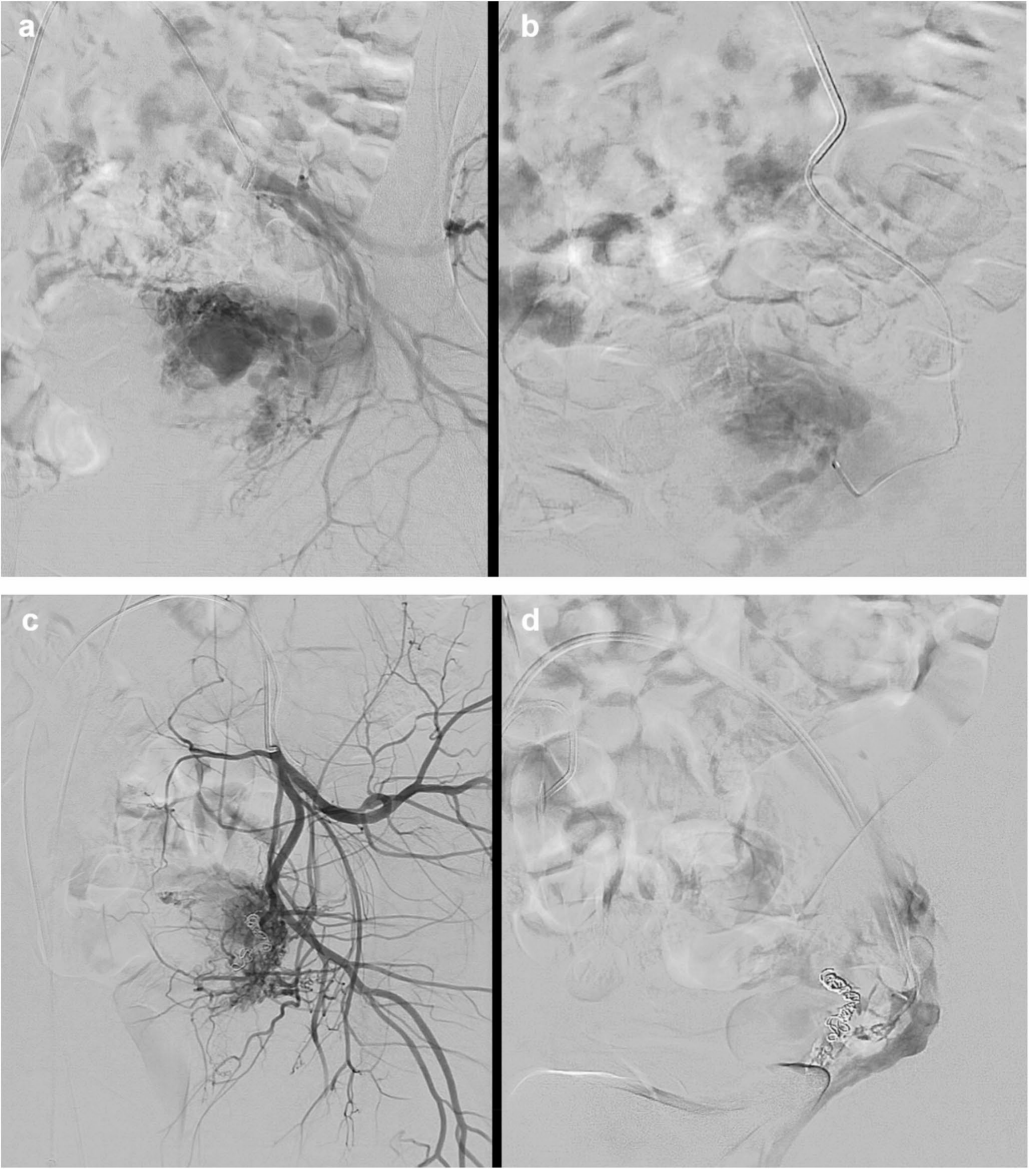
A 33-year-old female with significant bleeding post dilation and curettage (D&C) for miscarriage. Selective left uterine angiogram demonstrated UAVM with early venous filling (**a**). Post-gelatin embolisation angiogram showed much reduced contrast filling (**b**) of the vascular abnormality. This patient presented 33 days later with recurrent vaginal bleeding. The repeated selective uterine angiogram confirmed recurrent UAVM (**c**). This was successfully embolized with micro-coils and sodium tetradecyl sulphate (**d**)

A total of 8 UAE procedures were performed by 6 different interventional radiologists on these 7 patients, with 1 patient requiring a repeat UAE. In the initial UAE procedures, 6/7 patients underwent UAE with absorbable haemostatic gelatin, and 1/7 patient underwent UAE with PVA particles through supra-selective percutaneous microcatheters into branches of uterine arteries supplying the UAVM. Their digital subtraction angiography (DSA) confirmed the presence of UAVM. The vaginal bleeding ceased immediately in all 7 initial UAE procedures. (Figure 1b and c) However, 1 patient with absorbable haemostatic gelatin embolisation experienced recurrent bleeding 33 days later. Therefore, the overall clinical success rate of absorbable haemostatic gelatin in the UAE was 5/6 (83.3%). The 1/6 absorbable haemostatic gelatin patient who had recurrent bleeding was due to a massive complex vascular UAVM (Fig. 2a), and required a repeated UAE in which a combination of sodium tetradecyl sulphate injection and microcoils was used to achieve haemostasis.

All 7 patients remained free of complications, with no evidence of uterine infarcts or necrosis following embolisation, pulmonary embolism or, groin haematoma. The follow-up period for all these patients ranged from 23 to 77 months. Additionally, the postoperative length of hospital stay for patients who underwent absorbable haemostatic gelatin embolisation ranged from 1 to 2 days. In contrast, the 2 patients who had UAEs in whom permanent agents, including PVA particles and microcoils, were used had a hospital stay of 2 to 3 days. 3/6 patients (50%) subsequently became pregnant after absorbable gelatine UAE embolisation. The time to pregnancy ranged from 19 to 36 months post UAE.

## Discussion

There is a wide range of embolic agents that are used by interventional radiologists during embolisation procedures, which include temporary agents, such as absorbable haemostatic gelatin, and permanent agents, including PVA particles, coils, plugs, or liquid agents such as N-butyl-2-cyanoacrylate and sodium tetradecyl sulphate injection, and/or a mix of embolic agents [9]. Our study demonstrated that the use of purely absorbable haemostatic gelatin in the UAE is proven to be beneficial and risk-free in treating vaginal bleeding complications associated with acquired UAVM. It appears to offer a high clinical success rate while preserving fertility function, which is a major advantage over the surgical approach. The advantage of the gelatin is based on its temporary haemostatic effect, which gradually dissolves and is absorbed over time [10]. Its biodegradable nature allows for the successful obliteration of the AVM while minimising potential long-term uterine necrosis that could impact future placental implantation or fetal development [11].

The clinical success rate with absorbable haemostatic gelatin embolisation is 83.3% in our study, which is concordant with the published success rates of 88.4–94.1% in the literature that used a wide range of embolic agents [9, 12]. A systematic review by Ruiz Labarta et al. in 2022, which included 371 patients, reported a primary success rate of 79.2% [9]. This systematic review included studies utilising mixed materials (52.3%), including PVA particles, liquid agents, and other unspecified embolic agents; however, no absorbable haemostatic gelatin UAE was included. The available literature on absorbable haemostatic gelatin embolisation alone in the treatment of uterine AVM is relatively scarce. Camacho et al. reported a 94.1% success rate, which included 17 patients between 2013 and 2018, on the efficacy of gelatin UAE for acquired UAVM [12]. Our clinical success rate is close to that published by Camacho.

Our study showed that 3/6 patients (50%) subsequently became pregnant following gelatin UAE, with an average time to pregnancy being 19 to 36 months after embolisation. All 3 patients had successful full-term pregnancies, which suggested the presence of sufficient uterine blood flow to support a full-term pregnancy despite previous gelatin UAE. Other studies revealed an uncomplicated pregnancy rate of 41.2–77% [9, 12]. Ruiz Labarta et al. reported a major complication rate of 1.6%, including pulmonary embolism with embolic agent migration into the systemic circulation [9]. No pulmonary embolism or other complication was observed in our study.

Nevertheless, repeat embolisation utilising additional permanent embolic agents, such as microcoils, may be necessary in patients with larger or more complex vascular lesions [12]. These permanent agents help achieve more permanent occlusions, leading to a significant decrease in vascularity to the UAVM and thereby controlling uterine haemorrhage. In our study, 1 case with a complex vascular lesion developed recurrent bleeding and therefore required repeat embolisation with microcoils and sodium tetradecyl sulphate injection to achieve optimal results. This approach aligns with the findings of Wang et al., which included 11 out of 42 patients with large arteriovenous fistulae that underwent microcoil embolisation [13]. It demonstrated that a more aggressive approach with microcoils is preferred in cases of large arteriovenous fistulae encountered during the procedure, which provides long-term durability that could prevent recurrent haemorrhage [14].

This study also supports the premise that the UAE, with absorbable haemostatic gelatin, necessitates shorter hospital stay compared to hysterectomies. In our study, the mean post-procedural length of hospital stay for patients who underwent absorbable haemostatic gelatin embolisation was 1 to 2 days. In Kim et al., the average hospital stay following hysterectomy was 4 to 8 days for laparoscopic and open abdominal hysterectomy [15]. Hysterectomy poses a higher risk of complications, including postoperative infection in 9–13% of patients, venous thromboembolism in 1–12% of patients, genitourinary injuries in 1–2% of patients, bleeding complications, dehiscence, and neuropathy [16]. These emphasise the advantages of shorter hospitalisation and fewer complications associated with gelatin UAE compared to hysterectomy. Additionally, the shorter length of hospitalisation generally reflects quicker recovery time and minimises the risk of in-hospital complications [17].

Several limitations were observed in this study. Given the rarity of UAVM, this study had a relatively small cohort size. The findings may not be representative of the larger population. Since this was a single-institution case series study, the results might not be representative of other institutional settings, which may have different patient demographics, clinical practices, and healthcare systems. As such, future multi-institutional collaborations or diverse patient recruitment would be beneficial in comparing outcomes and complications of various treatment options. Another limitation of this study is the lack of documentation of any menstrual cycle changes post-UAE, as there may be some degree of blood supply alteration to the uterus that could influence menstrual cycles. Any alterations in menstrual function may go undetected. Future research incorporating menstrual cycle data to provide more comprehensive outcomes and the impact of absorbable haemostatic gelatin embolisation on reproductive health may be considered.

## Conclusion

Absorbable and biodegradable haemostatic gelatin, used as a temporary embolic agent, appears safe and effective in treating the majority of UAVMs in patients presenting in emergency settings, with a clinical success rate of 83.3%. This intervention not only preserves the uterus but also reduces the post-procedural length of hospital stay. Additionally, this approach allows for the possibility of successful pregnancy outcomes. In cases of large UAVM, embolisation using other adjunct embolic agents, such as coils or sodium tetradecyl sulphate, may need to be considered to achieve optimal results.

## Data Availability

The data that support the findings of this study are available from the first author and corresponding author upon reasonable request.
